# Assessment of the Pozzolanic Activity of a Spent Catalyst by Conductivity Measurement of Aqueous Suspensions with Calcium Hydroxide

**DOI:** 10.3390/ma7042561

**Published:** 2014-03-28

**Authors:** Sergio Velázquez, José M. Monzó, María V. Borrachero, Jordi Payá

**Affiliations:** 1 Facultad de Ingeniería, Universidad Panamericana Campus Guadalajara, Prolongación Calzada Circunvalación Poniente No. 49, Zapopan 54010, Mexico; 2 Instituto de Ciencia y Tecnología del Hormigón (ICITECH), Universitat Politècnica de València, Camino de Vera s/n, Valencia 46071, Spain; E-Mails: jmmonzo@cst.upv.es (J.M.M); vborrachero@cst.upv.es (M.V.B.); jjpaya@cst.upv.es (J.P.)

**Keywords:** spent fluid catalytic cracking catalyst, pozzolanic activity, electrical conductivity measurement, pozzolan/calcium hydroxide aqueous suspension

## Abstract

The pozzolanic activity of the spent catalyst produced by fluid catalytic cracking (FCC) has been studied by various methods in recent years. However, no quick and easy method has been reported for this activity based on the associated studies. In this work, the pozzolanic activity of a spent catalyst was investigated by measuring its electrical conductivity in aqueous suspensions of pozzolan/calcium hydroxide. The behavior of the FCC catalyst residue was compared to that of reactive and inert materials of similar chemical compositions. Further, the influence of temperature on the suspension was studied, and also, a new method was proposed in which the pozzolan/calcium hydroxide ratio was varied (with the initial presence of solid Ca(OH)_2_ in the system). It was concluded that the method is effective, fast and simple for evaluating the high reactivity of the catalyst. Therefore, this method is an alternative for the evaluation of the reactivity of pozzolanic materials.

## 1. Introduction

The study of the feasibility of using alternative materials in the construction industry has been increasing in recent years. A key factor for this increase has been the interest in promoting sustainable building practices. Pozzolanic materials have proven to be alternative materials that meet the requirements of sustainability; this is because they allow the incorporation of waste in the manufacturing of concrete. In addition, the partial replacement of cement by such material causes less demand for Portland cement, reducing quarrying, fuel consumption and CO_2_ emissions. It is widely known that pozzolanic materials can improve many properties of hydraulic binders made with them, such as mechanical strength, workability and various aspects related to durability. Several methods have been developed to assess pozzolanic activity. Many of these methods are based on the measurement of a property that is directly related to concrete applications, for example, the development of compressive strength, which entails the use of a significant study time to obtain adequate results. According to Donatello *et al.* [1], the methods for assessing pozzolanic activity can be categorized as either direct or indirect methods. Electrical conductivity measurement in liquid suspensions is considered an indirect method.

Raask and Bhaskar [2] were the pioneers in developing a methodology based on the measurement of electrical conductivity to assess the pozzolanic activity of fly ash. They conducted conductivity measurements in suspensions of fly ash in a 0.1 M hydrofluoric acid solution. From these data and the definition of a pozzolanic index, they classified fly ashes according to their reactivity.

The use of methods based on the electrical conductivity of an aqueous suspension of pozzolan/calcium hydroxide has significantly reduced the time needed to characterize these materials from the perspective of pozzolanicity [3–15]. The method of Luxán *et al.* [3] consists of the measurement of changes in the electrical conductivity in an aqueous suspension of calcium hydroxide (CH) with natural pozzolanic materials. The following are some of the most significant contributions offered by the method:

- The reaction between an aqueous suspension of CH and a pozzolanic material results in a decrease of the electrical conductivity of the solution, due to the fixation of CH by the pozzolan.
- Only two minutes are needed to assess the pozzolanic reactivity of natural materials, which allows for their classification as a function of conductivity variation, to be performed in accordance with the proposed method.
- The method is only applicable to natural pozzolanic materials, such as opal rocks or diatomaceous earths (diatomites); further, the method did not provide good results in assessments of the pozzolanic activity of artificial materials, such as fly ash (FA).

Feng *et al*. [4] used Luxán’s method to study the pozzolanic activity of rice husk ash. The study was conducted comparing the performance of rice husk pre-treated with hydrochloric acid and rice husk without this pretreatment. The ashes are subsequently obtained at different burning temperatures. They concluded that the ash obtained using pre-treated husks with HCl improves its pozzolanic activity markedly, and this behavior is observed for all studied burning temperatures.

Tashiro *et al.* [16] measured the electrical resistivity in pozzolan-lime pastes to evaluate the pozzolanic activity of various materials, such as fly ash, silica fume, kaolin, acid clay and zeolite. This method used steam curing at 70 °C, and 72 h were required for the assay.

McCarter and Tran [17] proposed a pozzolanicity index, which depends on the rate of change of the electrical conductivity and the time at which this parameter is maximum. For this study, they used lime-pozzolan pastes, investigating the pozzolanic behavior of microsilica, pulverized fly ash, metakaolin and calcined shale. In this case, the test was carried out in 48 h.

Payá *et al*. [5] modified Luxán’s method for application to the assessment of the pozzolanic reactivity of FA. The modifications are summarized here:

- Luxán proposed the use of a solution saturated in CH as a reagent for pozzolan. However, the preparation of a solution saturated in CH is a complicated process, because the dissolution rate of CH is very low at 40 °C. Consequently, several days are required to reach the saturated equilibrium point. Hence, the modification applied was to work with an unsaturated solution of calcium hydroxide with a concentration of 800 mg per liter of distilled water.
- Luxán established the classification of a pozzolanic material from the perspective of its pozzolanic activity, according to the change in conductivity of the suspension between the beginning and the end of the experiment (120 s). However, the author did not take into account the presence of soluble salts from the material studied in water, likely because the contribution to the conductivity of the natural pozzolans studied was negligible. However, this is not true when studying the pozzolanic activity of fly ash (FA) or rice husk ash (RHA) [15], due to the presence of a relatively high content of soluble salts in the pozzolanic material particles, deposited mostly on the surface. Based on the above, the proposed modification was to consider the contribution to the electrical conductivity from the solution of soluble salts that is derived from the pozzolanic material.

In addition to the measurement of electrical conductivity, these authors monitor the evolution of the pH of fly ash aqueous suspensions, comparing ashes with low lime content (class F) to ashes with high lime content (class C). The authors indicate that, in the latter case, given the contribution of lime by the pozzolan, it is not possible to use the proposed method, because an excess of lime is generated. As a result, the conductivity loss calculation becomes unreasonable.

Uzal *et al.* [6] studied the pozzolanic activity of clinoptilolite by measuring the electrical conductivity using the method proposed by Payá *et al.* [5], although the authors extended the test to four hours in duration. They studied pozzolan/calcium hydroxide suspensions with unsaturated solutions of calcium hydroxide, comparing the pozzolanic behavior of clinoptilolite with other pozzolans, such as silica fume, fly ash and a natural zeolitic pozzolan. The authors found that clinoptilolite has a high reactivity comparable to that of silica fume. They also studied the evolution of pH in aqueous suspensions of pozzolans, measuring an increase in pH due to the ions dissolved by pozzolans; this behavior corroborates the increase in electrical conductivity observed in these aqueous suspensions.

Sintharworn *et al.* [7] studied pozzolanic activity by measuring the electrical conductivity of various materials, such as silica fume, metakaolin and rice husk ash with aqueous suspensions at a temperature of 80 °C. They performed this study with aqueous suspensions by mixing ordinary Portland cement with the specific pozzolanic material of study. The trial time varied from five hours for metakaolin to 28 h for fly ash. In a later work [8], the same authors studied pozzolanic activity by measuring the electrical conductivity of suspensions with silica fume. In this work, the authors analyzed the influence of the calcium hydroxide concentration in the suspension, by studying unsaturated and saturated calcium hydroxide solutions, as well as a solution of an aqueous suspension of Portland cement. The study was conducted for three different aqueous suspension temperatures, including 40, 60 and 80 °C. The authors found that, by raising the temperature, the speed of the pozzolanic reaction was increased. They concluded that 80 °C is the ideal temperature for a rapid assessment by measuring the electrical conductivity (specifically, the authors reported a method that required seven hours to complete).

In several studies, Frías and Villar-Cociña [9–11] have used the electrical conductivity approach to study the behavior of several pozzolans (*i.e.*, sugar cane bagasse ash, bamboo leaf ash and natural rock zeolite) in aqueous suspensions. The method can require from 50 h [9,10] to 360 h [11]. In these works, the authors calculated the kinetic parameters of the pozzolanic reaction and incorporated the parameters into a kinetic-diffusive model. Their results showed that the kinetic parameters applied to their model are highly correlated with the experimental data.

Tironi *et al.* [12] studied the pozzolanic behavior of five calcinated natural kaolinitic clays. Their method is a modification of Luxán’s proposal of measuring the electrical conductivity of saturated calcium hydroxide aqueous suspensions. The modifications include the testing time (24 h) and the proportion of pozzolan/calcium hydroxide in solution.

Walker *et al.* [13] studied the pozzolanic behavior of several artificial pozzolans by various methods, including a method based on the measurement of electrical conductivity in saturated lime solutions, with the trials lasting approximately 150 h. The authors correlate the evolution of the electrical conductivity of the suspensions of these materials with different phases of the pozzolanic reaction, indicating that, during the first phase, the dominant factor of the pozzolanic activity is determined by its amorphous content, followed by its specific surface area.

The spent catalyst from fluid catalytic cracking (FCC) is a residue produced in the oil industry (1100 t/day [14]). Several studies have been performed on its pozzolanic activity, further demonstrating its high reactivity. These studies have been carried out by analyzing the evolution of the mechanical strength of mortars and concretes [18,19], thermogravimetric methods [20], as well as evaluating the advancement of the pozzolanic reaction with hydrochloric acid in cold temperatures [21]; however, the use of electrical conductivity to characterize the pozzolanic activity of a catalyst has not been reported yet.

In the present study, electrical conductivity and pH measurements were used to determine the pozzolanic activity of the FCC in aqueous suspensions of pozzolan/calcium hydroxide. The influence of the suspension temperature, as well as the pozzolan/calcium hydroxide ratio was also studied. In previously reported results, saturated or unsaturated Ca(OH)_2_ solutions were used; however, in them, no solid Ca(OH)_2_ was initially in the system, and consequently, there was a limit in the quantity of one of the reagents in the pozzolanic reaction. Here, a new method is also presented in which solid Ca(OH)_2_ was present in the initial system.

## 2. Results and Discussion

The assessment of the pozzolanic activity of mineral substances by measuring the electrical conductivity in aqueous suspensions takes into account the fact that the OH^−^ and Ca^2+^ ions dissolved in water react with the silica and alumina from the pozzolan to produce insoluble products, such as calcium silicate hydrate (CSH), calcium aluminate hydrate (CAH) and calcium aluminosilicate hydrate (CASH). The decline of these ions in solution decreases the electrical conductivity of the solution, if the solution is unsaturated with respect to CH. Because the pozzolanic reaction causes a reduction in the concentration of OH^−^ ions, a decrease in the pH of the system also occurs. When the pozzolan is added, the conductivity of the solution can increase, due to the presence of soluble salts in the material; such salts include sulfates, alkali halides and alkaline-earth halides. Therefore, it is necessary to determine the contribution of these salts (Section 2.1). In unsaturated solutions of CH, no solid CH is present; hence, the pozzolanic reaction causes a net decrease in the electrical conductivity of the suspension upon the addition of the pozzolan (Section 2.2). The pozzolanic reaction is also affected by the temperature of the medium; therefore, the reactivity at different temperatures can be evaluated, as well (Section 2.3). Finally, a new method based on electrical conductivity measurements is proposed and developed: in CH saturated systems, in which solid CH co-exists, the reaction of OH^−^ and Ca^2+^ ions can be compensated for by the dissolution of more calcium hydroxide, so that two competing processes coexist in the medium, that is, the fixation of ions and the dissolution of CH (Section 2.4). All of these aspects are discussed below.

### 2.1. The Solubility of Pozzolans in Water

**Figure 1.**
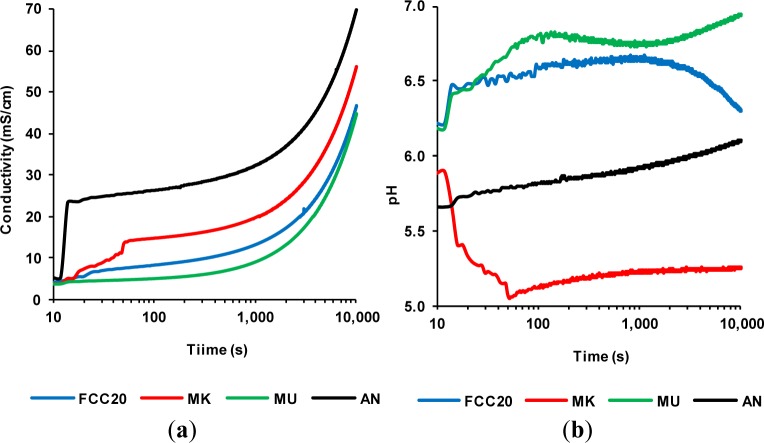
The contribution of the salts present in the pozzolanic materials to the (**a**) electrical conductivity of the water and (**b**) pH. FCC, fluid catalytic cracking. MK, metakaolin; MU, mullite; AN, andalusite.

To measure the contribution of the soluble substances present in pozzolans to the electrical conductivity of the water, the conductivity and pH of aqueous suspensions of FCC20, metakaolin (MK), mullite (MU) and andalusite (AN) were measured in the absence of calcium hydroxide (in accordance with the conditions of Experiment 1, Section 3.1). Figure 1a shows the variation over time of the electrical conductivity of the aqueous suspensions at 40 °C for all of these mineral additions. It can be observed that the contribution to the electrical conductivity of aqueous solutions by the salts present in the pozzolanic materials is very low. The values of electrical conductivity at 1000 s and 60 °C relative to the silica fume (SF) and the fly ash (FA) can be compared; in these cases, the values were approximately 400 μS/cm for SF and 2400 μS/cm for FA [5]. The value registered for rice husk ash (RHA) at 10,000 s and 40 °C was 200–1800 μS/cm [15]. The conductivity values obtained for the materials in the present study at 10,000 s and 40 °C were FCC20 = 46.6, MK = 56. 0, MU = 44.7 and AN = 69.7 μS/cm. This behavior suggests that the ionic contribution of the mineral additions used in this study is practically negligible. Therefore, based on these results, it was decided that the conductivity values in subsequent experiments would not be corrected, because the contribution of the typical salts of pozzolanic materials to the electrical conductivity was not relevant. This happens because unsaturated calcium hydroxide solutions (as discussed in the following sections) have a conductivity that, depending on the conditions of temperature and concentration, is in the range from 4300 to 10,000 μS/cm. Figure 1b shows a plot of the pH variation over time for the same suspensions of mineral additions to water. It can be observed that the obtained values of pH are neutral or slightly acidic. This behavior suggests that the neutralization of CH happens exclusively by pozzolanic processes, in which the OH^–^ ions will be fixed in the breaking processes of Si(Al)–O–Si bonds from the pozzolan.

### 2.2. The Evolution of Electrical Conductivity Loss at 40 °C in Unsaturated Pozzolan/CH Suspensions

In this test, the objective was to compare the behavior of FCC20 with various materials. As a pozzolanic material with a similar composition to the catalyst, MK was used. As inert materials, MU and AN were used. All of these materials were selected, because their chemical compositions are similar to that of FCC20. In the absence of solid calcium hydroxide, the total amount of calcium hydroxide is present as dissolved OH^−^ and Ca^2+^ ions and is available for the pozzolanic reaction. Therefore, in these trials, an unsaturated CH solution was prepared to study the pozzolanic reaction, in order to evaluate the net decrease in electrical conductivity [5]. A solution of 0.8 g/L was established (see Section 2.2, Experiment 2). This means that the concentration was below the maximum amount of CH that can be dissolved in water at that temperature (1.41 g/L) [22]. In this case, the pozzolanic reaction will result in a decrease in the conductivity and pH of the suspension, given that all of the calcium hydroxide is dissolved. Payá *et al.* [5] explain that, when there is an important contribution to the electrical conductivity by the pozzolan due to high salinity, this must be subtracted from the total electrical conductivity. They define (*C_xl_*)*_t_* as the conductivity of the pozzolan/CH suspension at a given time “*t*” and (*C_x_*)*_t_* as the electrical conductivity of the aqueous suspension of the pozzolan in the absence of calcium hydroxide at a time “*t*”. Then, the authors obtain the absolute electrical conductivity of the pozzolan/CH suspension (*C_xa_*)*_t_*, which considers the correction due to dissolved salts as (*C_xa_*)*_t_* *=* (*C_xl_*)*_t_* − (*C_x_*)*_t_*. Based on these definitions, the authors calculate the percentage of electrical conductivity loss due to the fixation of lime by the pozzolan as:

$${\left(\%LC\right)}_{t}=\frac{C_{0}-{\left(C_{xa}\right)}_{t}}{C_{0}}100$$ (1)

where:

- (*%LC*)*_t_* is the percentage loss of electrical conductivity in a suspension after a given time “*t*”,
- *C*_o_ is the electrical conductivity of the solution of calcium hydroxide before the addition of the pozzolanic material, and
- (*Cxa*)*t =* (*Cxl*)*t −* (*Cx*) is the corrected value of the electrical conductivity of the pozzolan/CH suspension at a given time, “*t*”.

In the present study, it has been established that it is not necessary to correct the conductivity value due to the content of salts derived from the pozzolanic materials (Section 2.1); thus, (*C_xa_*)*_t_* ≈ (*C_xl_*)*_t_*.

**Figure 2.**
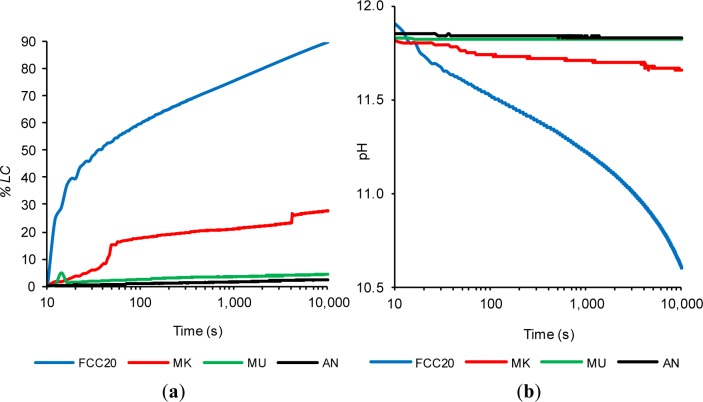
The time evolution for the pozzolan/Ca(OH)_2_ water suspensions (1 g/40 mg in 50 mL) at 40 °C of: (**a**) the loss of electrical conductivity and (**b**) pH. *%LC*, percentage loss of electrical conductivity.

Figure 2a shows plots of the loss of electrical conductivity (*%LC*)*_t_* curves, and Figure 2b presents plots of the pH curves. Regarding the inert materials (MU and AN), it can be observed that the values of (*%LC*)*_t_* are extremely low (the values after 10,000 s are only 4.2% and 2.3%, respectively, as shown in Figure 2a), which indicates negligible pozzolanic activity. This behavior coincides with the observed values of pH variation for the same materials (see Figure 2b), which remain practically constant throughout the trial period. The MU suspension starts with a pH of 11.83 and ends with a value of 11.82 at 10,000 s, while the conductivity of the AN suspension varies from 11.85 to 11.83. The inert behavior of these materials is in significant contrast to the highly reactive behavior of FCC20. In Figure 2a, it can be observed that the (*%LC*)*_t_* for FCC20 reaches high values, namely, 60.0% at 100 s, 75.8% at 1,000 s and 90.0% at 10,000 s. This high loss of electrical conductivity for the FCC20 is related to the high degree of fixation of the Ca^2+^ and OH^−^ ions. The fixing of OH^−^ ions leads to a large decrease in pH (as can be observed in Figure 2b). The pH values of the FCC20 suspension are 11.90 at time zero, 11.52 at 100 s, 11.22 at 1000 s and, finally, 10.60 at 10,000 s. Finally, in these trials, it can be observed that MK also shows a high degree of pozzolanic activity, although this reactivity is not as large as that recorded for the FCC20 during this study. When comparing these pozzolans, it is remarkable to note that the pozzolanic reaction is immediate for the catalyst, which does not happen for MK; this observation further corroborates the results observed by thermogravimetric analysis [20]. For the MK suspension, a slight amount of initial activity can be observed, which accelerates at approximately 40 s and slows down until 4100 s is reached, at which point, a second acceleration can be observed. For comparative purposes with FCC20, the values of (*%LC*)*_t_* for MK are 17.59% at 100 s, 21.03% at 1000 s and 27.59% at 10,000 s. This slower evolution of the pozzolanic activity of MK is corroborated by the variation in pH values observed in Figure 2b, where a slight decline can be observed from the initial pH value of 11.81 to 11.66 after 10,000 s. These results suggest a significantly lower consumption of hydroxyl ions by MK in comparison to FCC20, which exhibited a small drop in pH of 0.15 for MK, while the net decrease for FCC20 was 1.3.

### 2.3. The Influence of Suspension Temperature on the Pozzolanic Activity

**Figure 3.**
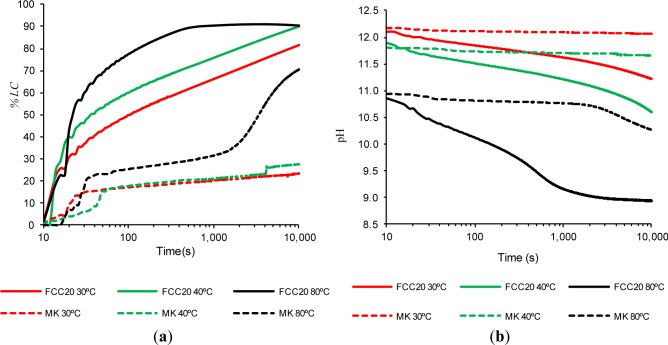
The time evolution for the pozzolan/Ca(OH)_2_ water suspensions (1 g/40 mg in 50 mL) of: (**a**) the loss of electrical conductivity and (**b**) pH. The influence of water temperature.

The chemical reaction between calcium hydroxide and the pozzolanic material is influenced by the temperature of the suspension, which directly affects the reaction’s degree. Therefore, this experiment was developed to study the influence of the suspension temperature on the pozzolanic reaction. These experiments were conducted by comparing only the FCC20 to the MK, given that the previous experimental results showed that the inert materials (MU and AN) behave as such when assessed based on electrical conductivity (see Section 2.2). The temperatures considered in the experiment were 30, 40 and 80 °C. Initially, the concentrations of the Ca^2+^ and OH^−^ ions are maintained (see Section 3.3, Experiment 3). Although the conductivity of each suspension increases with temperature [5], it was not necessary to make any correction given that the (*%LC*)*_t_* value is obtained as a ratio of conductivity data. Given that the results at 40 °C showed very high catalyst reactivity, the evolution of the loss of conductivity at two additional temperatures, 30 and 80 °C, was identified for further investigation, as well as the repetition of these experiments with MK as a comparison. The initial solutions of CH remained unsaturated at the three temperatures tested (the saturated concentrations of the aqueous solution of calcium hydroxide are 1.52 g/L at 30 °C, 1.41 g/L at 40 °C and 0.98 g/L at 80 °C [22]). Figure 3a shows the results for the loss of electrical conductivity percentage, when the ratio of pozzolan/CH used (1 g/40 mg in 50 mL of water) was held constant for the three temperatures studied. Figure 3b shows the evolution of the pH values for these same assays. Figure 3a shows that, for both pozzolans, increasing the water temperature of the suspension from 40 to 80 °C significantly increases the (*%LC*)*_t_* value. For FCC20, it can be observed that, at 80 °C, the (*%LC*)*_t_* reaches values above 90% at 660 s, which is the maximum (*%LC*)*_t_* value reached after 10,000 s in the 40 °C trial. After this time, the FCC20 fixates a minimal amount of additional calcium hydroxide; this result might be due to a coating on the catalyst’s surface of the products of hydration and, perhaps more importantly, due to the negligible amount of available dissolved calcium hydroxide. The (*%LC*)*_t_* values for FCC at 80 °C at 100, 1000 and 10,000 s are, respectively, 77.4%, 90.4% and 90.4%. The behavior of FCC20 at 80 °C is consistent with the measured pH values (see Figure 3b), which show a significant decrease between the beginning of the trial (pH value of 10.86) to values of approximately 9.17 after 1000 s, followed by a slight additional decrease to 8.94 after 10,000 s. This confirms that, in the final period of time under investigation, the concentration of the ions remains practically unchanged. When the temperature is reduced from 40 °C to 30 °C, it is noted that, for both pozzolans, the values of (*%LC*)*_t_* decrease slightly. For FCC20, the behavior is practically the same as that measured at 40 °C, with a difference in the (*%LC*)*_t_* values of approximately 10%. The (*%LC*)*_t_* values after 100, 1000 and 10,000 s are, respectively, 49.9%, 66.3% and 81.8% (see Figure 3a). This decrease in the reactivity of FCC20 when reducing the temperature from 40 to 30 °C can also be observed in the variation of pH values, corroborating the parallel behavior observed in both trials. The pH values range from 0.20 to 0.63 throughout the whole test (see Figure 3b).

Unlike FCC20, for the MK test at 80 °C, both processes observed for the fixing of calcium hydroxide in the test at 40 °C are still observed (see Figure 3a). The first of these processes is observed at approximately 40 s, where the (*%LC*)*_t_* increases from approximately 17% to 25%. During the second stage, from approximately 2,000 s to the completion of the test (10,000 s), an increase of the (*%LC*)*_t_* is observed from 37.5% to 70.7% (the maximum (*%LC*)*_t_* value at 40°C was only 27.6%). Regarding the pH values recorded for MK at 80 °C (see Figure 3b), only the second stage can be clearly observed for the same period in which the (%*LC*)*_t_* was measured, *i.e.*, a significant decrease in the pH values from 10.73 to 10.28, between 2000 and 10,000 s. Based on the sharp rise in (*%LC*)*_t_* values and the decrease in pH values, it can be concluded that MK takes longer to react than FCC20, which can be compensated for by the application of thermal activation. When the test temperature is reduced from 40 to 30 °C for MK, the (*%LC*)*_t_* values remain essentially constant, and the second stage is not observed in the test carried out at 30 °C. It is possible that a longer test for MK suspensions at 80 °C could reach the same (*%LC*)*_t_* values obtained with FCC20 (at any of the three temperatures studied, because they all seem to converge to the same value over time). However, it remains evident that, for short times, the behavior of FCC20 is far superior to that of MK, even when comparing high temperatures with MK to low temperatures with FCC (the (*%LC*)*_t_* value for the FCC20 after 10,000 s and 30 °C is 81.8%, while for MK after 10,000 s and 80 °C, the value is slightly lower at 70.7%).

### 2.4. The Influence of the CH/Pozzolan Ratio

**Figure 4.**
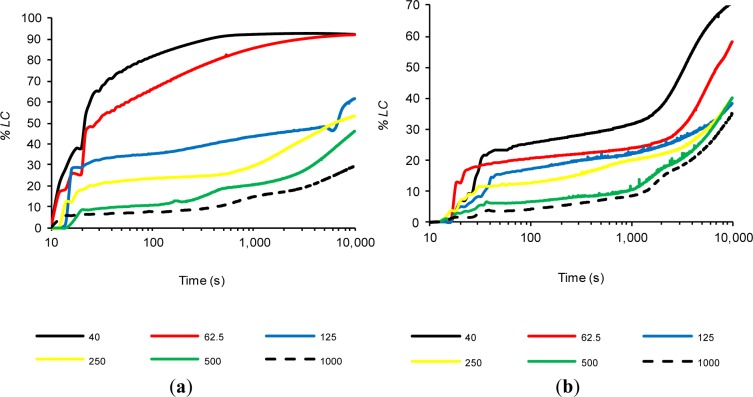
The time evolution of *%LC* for pozzolan/water suspensions (1 g/50 mL) when varying the amount of CH (mg) for: (**a**) FCC20 and (**b**) MK.

**Figure 5.**
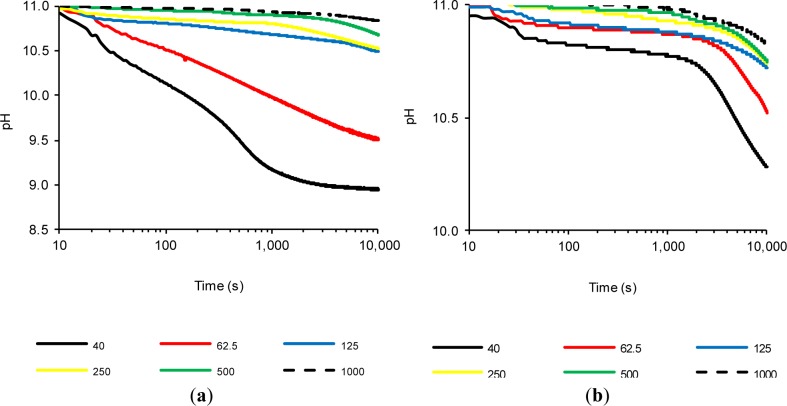
The time evolution of the pH for pozzolan/water suspensions (1 g/50 mL) when varying the amount of CH (mg) for: (**a**) FCC20 and (**b**) MK.

Previous tests confirmed that FCC20 consumes calcium hydroxide very quickly, because significant changes in the (*%LC*)*_t_* values were observed after a few seconds. Therefore, significantly lower variations were observed over time, as a result of the depletion of available calcium hydroxide, which had already reacted with the pozzolan (regardless of the test temperature). Therefore, it was decided to carry out a new method: a study in which the amount of calcium hydroxide was increased, while holding the amount of pozzolan added constant and performing the tests at 80 °C to obtain a faster pozzolanic reaction. Therefore, in this experiment, saturated solutions were also considered, where solid calcium hydroxide at 80 °C coexists. Due to the coexistence of solid CH and dissolved OH^−^ and Ca^2+^ ions in these assays, the objective was to study the pozzolanic reaction when two processes were present simultaneously, *i.e.*, ion fixing by the pozzolanic reaction and the dissolution of CH as a result of the loss of the saturation provoked by the pozzolanic reaction. Six suspensions were studied (Section 3.4, Experiment 4). Figure 4 shows the variation of (*%LC*)*_t_* over time for suspensions with both pozzolans (FCC20 and MK), and Figure 5 shows the evolution of pH over time for both pozzolans. For both pozzolans, it is evident that an increase in the CH/pozzolan ratio decreases the value of (*%LC*)*_t_*. For the spent catalyst (see Figure 4a), there is a significant decrease in the (*%LC*)*_t_* when the addition of calcium hydroxide is increased from 62.5 to 125 mg. This relationship (0.0625–0.125 g of CH/g of FCC20) is similar to the previously reported value [21] of the grams of calcium hydroxide fixed per gram of catalyst dosage between one and 14 days of curing for Portland cement pastes at a temperature of 20 °C (this value ranges from 0.05–0.08 g of CH/g of FCC20). The pozzolanic reaction is facilitated in this test by the use of an environment with a high proportion of liquid phase, a higher temperature and a pozzolan/CH system (without Portland cement), which explains why the values of the ratio are similar, even if the testing time is significantly lower (10,000 s). It is remarkable that, for tests with 125 mg or more of CH, the commencement of a second phase of pozzolanic activity can be observed for FCC20. This second stage has not been observed before, because the previous suspensions were always unsaturated. Under saturated conditions (for example, see the tests with 250, 500 and 1000 mg of CH), the first stage corresponds to a slight continuous increase in (*%LC*)*_t_* from the beginning of the test to approximately 1000 s. This increase is most likely due to the excess of CH, which increases the amount of dissolved ions that favor the pozzolanic reaction, but also causes a diminishing effect on the measurement of conductivity loss, given that new ions are continuously dissolved. That is, two processes are in competition, namely, the fixation of Ca^2+^ and OH^−^ ions by the pozzolan, as well as the dissolution of solid calcium hydroxide. A second stage is visible at approximately 1000 s; at later times, the loss of electrical conductivity accelerates. This result can be explained by assuming that the more soluble fraction of calcium hydroxide particles has already been dissolved; hence, the speed of the pozzolanic reaction tends to be more important than the dissolution rate of calcium hydroxide. With respect to the evolution of pH relative to the use of FCC20 in these tests (see Figure 5a), the significant decrease in pH is again clearly visible when progressing from a solution with 125 mg of CH to 62.5 mg, which is in accordance with the evolution of the electrical conductivity values of the suspensions.

Regarding the influence of the calcium hydroxide concentration on the behavior of MK, it can be observed that the calcium hydroxide concentration also presents a decrease in the (*%LC*)*_t_* values when the amount of CH in the suspension is decreased. However, the significant decline observed for FCC20 when changing the amount of CH from 62.5 to 125 mg is significantly less pronounced for MK. More generally, it can be said that, for all of the tests with MK, regardless of the availability of CH (saturated or unsaturated solutions), the (*%LC*)*_t_* values obtained are not high, most likely due to the long reaction times required for this pozzolan. It is also notable that the (*%LC*)*_t_* values at 10,000 s for MK suspensions with a CH amount of 125 mg or higher are practically identical (between 35% and 40%). With respect to the evolution of pH values over time for MK when varying the amount of CH (see Figure 5b), only changes in the tests with 40 mg of CH (unsaturated dissolution) and 62.5 mg of CH (saturated solution) are observed. For the CH-saturated systems, the behavior observed in the conductivity is corroborated, in the sense that at 10,000 s, all of the solutions tend to approximately the same value of pH (between 10.72 and 10.83), as well as the fact that the decrease in pH values was very low, because all of the tests began with a value of approximately 11.00.

## 3. Experimental Section

Aqueous suspensions of calcium hydroxide and the pozzolan in question were used for this study. The calcium hydroxide used was 96% pure, obtained from Riedel de Haën. The catalyst residue used from catalytic cracking process was supplied by BP OIL Spain S.A. (Castellón, Spain). Metakaolin (MK, Metastar, ECC International, St Austell, UK) was the pozzolanic material used for comparison. The chemical compositions of the FCC and MK are shown in Table 1. The catalyst was ground before use for 20 min (FCC20) with a laboratory mill (Gabrielli Mill-2, Gabbrielli Technology srl, Calenzano, Italy) to activate its pozzolanic behavior [23]. Two inert crystalline materials, mullite (Al_6_Si_2_O_13_) and andalusite (Al_2_SiO_5_), were used to establish a comparison for the FCC20 pozzolanic behavior. The percentages of aluminum and silicon oxides in these materials are, respectively, 71.8% and 28.2% for mullite (MU) and 63% and 37% for andalusite (AN). The average particle diameters of the four materials studied are: FCC20 = 19.96 μm; MK = 5.84 μm; MU = 37.36 μm and AN = 31.05 μm.

The pozzolanic activity was measured based on the loss of electrical conductivity, according to the method described by Payá *et al.* [5], which uses a conductivity-meter (Crison microCM2201, Crison Instruments S.A., Alella, Spain and a pH-meter (Crison micropH 2001, Crison Instruments S.A.), both with an Recommended Standard 232 (RS232) output. The experiments were performed using a thermally insulated reactor for the temperature regulation. Additionally, the experiments were carried out in isolation to prevent carbonation. Four types of experiments were conducted.

### 3.1. Experiment 1

The electrical conductivity and pH of aqueous suspensions of FCC20, MK, MU and AN in the absence of calcium hydroxide were measured. One gram of pozzolan was added to 50 mL of distilled water into a thermostatic vessel at 40 °C. Then, the electrical conductivity and pH measurements were recorded for a total period of 10,000 s.

### 3.2. Experiment 2

This experiment was performed at 40 °C. The procedure was as follows: an unsaturated solution of calcium hydroxide was prepared by dissolving 40 mg of CH with 50 mL of distilled water in a closed thermostatic vessel to prevent carbonation and then raising the system temperature to 80 °C to accelerate dissolution (which requires approximately one hour). Subsequently, the temperature was decreased to 40 °C, and 1 g of the mineral under study was added (either FCC20, MK, MU or AN, Sibelco Minerales S.A., Nules, Spain). Next, the electrical conductivity and pH of the solution was immediately measured for a total time of 10,000 s.

### 3.3. Experiment 3

These assays were performed as follows: unsaturated solutions of calcium hydroxide were prepared following the same procedure described in Section 3.2. Then, the temperature was set to the corresponding test value (30, 40 and 80 °C). One gram of the pozzolan under study was immediately added (FCC20 or MK), and then, the electrical conductivity and pH were measured for a total time of 10,000 s.

### 3.4. Experiment 4

The procedure of this test was carried out by adding a pre-set amount of calcium hydroxide, namely, 40.0, 62.5, 125.0, 250.0, 500.0 or 1000.0 mg, to 50 mL of distilled water in a thermostatic vessel at a temperature of 80 °C. Of all assays, only that with 40 mg yielded an unsaturated calcium hydroxide solution. Initially, the five remaining suspensions were saturated at a temperature of 80 °C with an excess of solid CH (based on the fact that, at 80 °C, saturation is reached at 0.98 g/L [22]). After an hour of maintaining the solution under magnetic stirring at 80 °C, 1 g of pozzolan (FCC20 or MK) was added, and the pH and electrical conductivity were measured for 10,000 s.

## 4. Conclusions

Based on the study of the pozzolanic activity of a spent catalyst from fluid catalytic cracking (FCC20), evaluated by measuring the electrical conductivity in a calcium hydroxide aqueous suspension, the following can be concluded.

1) The method of Luxán, modified by Payá *et al.* [5], was used without the need to correct the electrical conductivity values due to the contribution of salts by the spent catalyst, given that the contribution of the pozzolan to this property is negligible.
2) The (%*LC*)*_t_* of the calcium hydroxide suspension with FCC20 is far superior to that of MK, MU and AN, yielding a value of almost 90% (three times higher than that of MK) at 40 °C and 10,000 s.
3) Increasing the reaction temperature of calcium hydroxide suspensions increases the (*%LC*)*_t_* significantly, with the effect being more pronounced for the catalyst when compared with MK.
4) With respect to the new proposed method (the presence of solid calcium hydroxide), when increasing the amount of calcium hydroxide present in the suspension for the same amount of pozzolan, the value of (*%LC*)*_t_* decreases significantly.
5) For the test in which 62.5 and 125 mg of calcium hydroxide were mixed with FCC20 (1000 mg), it was observed that the consumption of ions by the pozzolanic reaction was always more important than the solution rate of CH. For the remaining tests using a higher amount of CH, only after measurements beyond 1000 s could this behavior be observed.
6) The results obtained by this method confirm the high pozzolanic reactivity of the catalyst. These reactivity data are consistent with those obtained in previous studies by other techniques, such as thermogravimetry and mechanical strength [18–21,23,24].

## Figures and Tables

**Table 1.** Chemical compositions of FCC20 and MK (wt%).

Pozzolan	Chemical compositions (wt%)							
	SiO_2_	Al_2_O_3_	Fe_2_O_3_	CaO	MgO	K_2_O	Na_2_O	LOI*
FCC20	48.2	46.0	0.95	<0.01	<0.01	<0.01	0.50	1.50
MK	52.1	41.0	4.32	0.07	0.19	0.63	0.26	0.60

* LOI: Loss on ignition
